# Magnitude and factors associated with postoperative depression among adult orthopedics patients during COVID-19 pandemics: A multi-center cross-sectional study

**DOI:** 10.3389/fpsyt.2022.965035

**Published:** 2022-07-29

**Authors:** Shimelis Seid Tegegne, Yewlsew Fentie Alle

**Affiliations:** Department of Anesthesia, School of Medicine, College of Health Sciences, Debre Tabor University, Debre Tabor, Ethiopia

**Keywords:** depression, magnitude, orthopedics procedures, postoperative period, psychological factors

## Abstract

**Background:**

Postoperative depression is one of the devastating problems and important health concerns in adult orthopedics surgical patients. It is often under-diagnosed and appropriate perioperative management of patients is recommended. This study aimed to determine the magnitude and factors associated with postoperative depression among orthopedics patients in Ethiopia.

**Materials and Methods:**

This multi-center cross-sectional study was conducted on 443 adult post-orthopedics surgical patients. All the data were entered and analyzed with SPSS version 25. Bivariable and multivariable logistic regression was used to identify the associated factors with the outcome variable. *P-*values <0.05 were taken as statistically significant with 95% CI. Data were collected after distributing 9-item standard patient health questionnaires and the Oslo-3 item social support scale tool.

**Result:**

Based on our study result, the magnitude of postoperative depression among adult orthopedics surgical patients was 61.8% (95% CI: 56.8–65.7). Using multivariable logistic regression analysis, factors which had an association with postoperative depression were female in gender, Farmer in occupation, having a history of previous substance use, history of anxiety, Patients who had moderate to poor social support, BMI <18.5 kg/m^2^, and patients who had an open fracture.

**Conclusion:**

The magnitude of postoperative depression was high. Due emphasis needs to be given to screening and treatment of postoperative depression, especially among patients of the female gender, farmer occupation, moderate to poor social support, history of substance use and anxiety, low BMI, and open fracture.

## Introduction

Orthopedic trauma is defined as an injury to parts of the musculoskeletal system such as bones, ligaments, and joints (1). More than 2.8 million people faced orthopedic injuries every year and it is an unforeseen life-changing event (2). After fracture; survivors commonly experience depression, anxiety, and post-traumatic stress syndrome (3, 4). The reports of different studies showed that 28–60% of hospitalized orthopedic patients experience psychiatric problems (5–7). The prevalence of psychological illnesses following traumatic injury surgery varies according to the types of trauma, the instruments used for measurement, and the timing of the assessment (8, 9). Depression is one of the most common mental disorders worldwide and is the second leading cause of disability (10) and a major contributor to suicide (11). Major depression is reported in 5–10% of older medical outpatients seeing a primary care, 8–16% of community-dwelling older adults, and 10–12% of medical-surgical hospitalized older adults (12).

More studies have been conducted on depression levels of the patient in Europe and the United States compared to other countries. A study done in the United Kingdom showed that among post-orthopedics surgical patients, 50% of the patients had depression symptoms (13). However, it has been stated that for patients undergoing orthopedic surgery, the functional recovery, complication, cost, and death are focused on, and less consideration is given to the psychological condition of the patients (14, 15). Despite the high burden of orthopedic trauma in Ethiopia, the psychiatric sequelae are almost unforeseen (16). This leads to avoidable but unalleviated suffering for the survivors.

Untreated postoperative depression may result in cognitive dysfunction, greater postoperative pain, progression of malignant tumors, poor health-related quality of life, post-operative infections, and other complications (17, 18). The interaction of surgery and anesthesia with depression may result in a significant increment in mortality and morbidity risks for patients. Major depressive disorder is a common complication after orthopedics surgery, which leads to further physical morbidity and mortality risks (19). A study showed that around 15% of patients who undergo surgery under anesthesia are at high risk of complications, which contributed to 80% of all perioperative deaths (20).

Depression after orthopedics surgery is under-diagnosed and undertreated in Ethiopia (18). Different factors contributed to the occurrence of postoperative depression. Older adults, men, patients from ethnic minority groups, and patients with medical comorbidities are particularly recognized as at high risk of depression (21). As different studies showed symptoms and frequency of depression differ between societies' backgrounds, and these are thought to be associated with cultural, genetic, and environmental factors (22). The depression levels of the patients, from different countries with various socio-cultural backgrounds, are also a controversial subject (23).

Effective management of depression before and after orthopedics surgery requires several steps. Some of them are; early detection and diagnosis, patient engagement and education in treatment, initiation of evidence-based pharmacotherapy, close follow-up focusing on treatment adherence, and treatment side effects (24).

Even though different researches were conducted regarding the magnitude of depression in the community people of Ethiopia, studies on depression among post-surgical patients were limited (25). This study aimed to assess the magnitude and factors associated with postoperative depression after orthopedics surgery among adult patients in Amhara regional comprehensive specialized Hospitals. This helps us to identify the current gap and to improve quality services.

## Materials and methods

### Study design and setting

After we got ethical approval from Debre Tabor University ethical review committee following the declaration of Helsinki, a multi-center cross-sectional study design was conducted on 443 orthopedics patients who underwent surgery from January 10/ 2021 to April 15 / 2021 in the Amhara regional CSHs. This study was already registered at researchregistry.com with a unique identifying number of 7493.

### Study areas

Amhara regional state is one of the regions among the eleven states in Ethiopia and it is located in North-West Ethiopia. Its capital city is Bahir Dar, which is 565 km far from Addis Ababa. There are seven comprehensive specialized hospitals in the Amhara regional state. These are; Debre Tabor Comprehensive Specialized Hospital, Felege Hiwot Comprehensive Specialized Hospital, Tibebe Gihon Comprehensive Specialized Hospital, Debre Markos Comprehensive Specialized Hospital, Dessie Comprehensive Specialized Hospital, Debre Birhan Comprehensive Specialized Hospital, and University of Gondar Comprehensive Specialized Hospital. Orthopedics surgery is performed on the daily basis and on average, around 1,000–2,000 orthopedics procedures have been performed every year in each CSHs.

### Study participants and data collection procedures

After obtaining ethical approval per the guidelines in the Declaration of Helsinki, written informed consent was taken from each study participant using their mother tongue language (Amharic). Full information regarding the benefit and risks of the study was disclosed to study participants. They were also informed of their full right to refuse the study. Confidentiality was assured by removing identifiers and locking the questionnaires after data collection in a secured area. The investigators developed structured questionnaires in the English language and, it was translated into their mother tongue language (Amharic). Eligible study participants completed an initial interviewer-administered questionnaire on the day before their surgery.

Regarding the occurrence of postoperative depression in orthopedics patients, a study done in the UK showed that the exact meantime point for the development of depression was 2.43 days (SD = 1.40 days) and the time of maximal postoperative depression was 2.93 days (SD = 1.72 days) (13). However, still, depression after surgery can occur at any moment in the postoperative period. By considering this, we have collected the data 1 day before surgery and during the time of discharge in the postoperative period.

The preoperative evaluation was taken as a baseline measurement to compare with their post-operative scores. No measurements were conducted on the day of surgery, as it was felt that results obtained on this day might not be an accurate reflection of how the patient was feeling. Subjects were then asked to complete the questionnaire during the postoperative discharge time.

### Inclusion and exclusion criteria

All eligible adult patients who underwent elective and emergency orthopedics surgeries in Amhara regional CSHs were included. These patients who had a preoperative psychiatric disorder, change in mentation, those who could not be contacted even after two visits, those who have preoperative depression, individuals having aphasia, disorders of hearing and speech, and those who had a frequent history of surgical exposure during the study period were excluded.

### Study variables

Dependent variable: Postoperative depression.

Independent variables**:** sociodemographic variables, BMI, Preoperative variables (history of substance use, anxiety, depression, social support, and pain level), type of surgery and anesthesia, site of surgery, type of fracture, PHQ-9 score, Oslo-3 point scale, and postoperative pain level.

### Sample size determination

There are no studies conducted in Ethiopia regarding the magnitude of postoperative depression among adult orthopedics surgical patients. So, the sample size was calculated by using a single population proportion formula.

Where n = is the desired sample size; *Z* α/2= is standard normal distribution usually set as 1.96 (corresponds to 95% confidence level); *p* = population proportio*n* = 0.5), and *d* = degree of accuracy desired (marginal error is 5% (0.05). So, *n* = 385.

Then when we add 15% of the non-response rate, the final sample size is *n* = 443

### Sampling techniques

We used a simple random sampling to collect data from eligible adult orthopedics surgical patients who have undergone surgery at the Orthopedics operation rooms of Amhara regional CSHs. After we prepared a list of patients (sampling frame), the data collectors choose a sample using a random number generator software. Then, data were collected in every constant step of the generated number. Comparable study participants were selected by considering the yearly case flows in each Comprehensive Specialized hospital. Of the total study participants, 19 patients were not included in the analysis due to incomplete data (as shown in Table 1).

**Table 1:** Shows total selected patients distribution across different Comprehensive Specialized Hospitals of Amhara regional state, in North-West Ethiopia (*n* = 443).

**List of Hospitals**	**Number of cases**	**Number of cases excluded from**	**Total samples analyzed**
	**included (*n*)**	**analysis due to different reasons**	***n* (%)**
Debre Tabor CSH	66	3	63 (14.20%)
Felege Hiwot CSH	62	1	61 (13.77%)
Tibebe Gihon CSH	64	2	62 (13.99%)
Debre Markos CSH	63	2	61 (13.77%)
Debre Birhan CSH	63	4	59 (13.32%)
University of Gondar CSH	64	5	59 (13.32%)
Dessie CSH	61	2	59 (13.32%)
Total patients	443 (100%)	19(4.3%)	424 (95.7%)

CSH, Comprehensive Specialized Hospital; n = number; %, percentage.

### Data collection tools

We used a patient Health Quality−9 scale (PHQ-9) and Oslo- 3 item social support scale to assess postoperative depression and social support levels respectively. PHQ-9 is a validated questionnaire, which has been designed to screen for perioperative depression in the general hospital setting. The PHQ-9 is a public domain instrument for depression screening (26). This instrument only takes 2–5 min to complete. The Cronbach's α coefficient of PHQ-9 is 0.892. It has shown more than 88% specificity and sensitivity for assessments of depressive disorders.

The PHQ-9 initially asks two questions about anhedonia and mood to identify if further screening should be done. By any of the following problems, how often have you been bothered? Then; Patients indicate their feeling as 0(not at all), 1(several days), 2(more than half the days), and 3(nearly every day). A score of 1–3 may show that the patient had minimal depression. A score of 3 or above needs further evaluation by asking the remaining questions to diagnose possible depression. A score of 0–4 is considered normal, 5–9 indicates mild depressive symptoms, 10–14 suggests moderate symptoms, and 15 or higher identifies those with severe depression. In our study, we diagnosed depression when the PHQ-9 score was above 4 and entered regression analysis.

Regarding patient social support level, data were collected by Oslo 3- item social support scale with a sum score scale ranging from 3 to 14. It has three broad categories: poor social support ranges from 3 to 8, moderate social support from 9 to 11, and strong social support ranges from 12 to 14 (27). Because of the widespread use of the instrument in different large-scale research settings, the Oslo social support scale is considered the measure of choice for different age groups of men and women in the current studies. It has a good validity test and internal consistency with an α of 0.640. The scale's brevity makes it a relatively low value (28).

### Data quality control

Pretest was done on 5% (22) of postoperative orthopedics patients. Training about the questionnaire was given to data collectors before the data collection period. Our data collectors were Anesthetists and they were 7 in number. One anesthetist was allocated for each comprehensive specialized hospital. The collected data was checked for its completeness and clarity on daily basis and corrections were made accordingly. Follow-up and supervision were done by the principal investigator throughout the study.

### Data analysis and interpretation

All the data were entered and analyzed with SPSS version 25. For this cross-sectional study, descriptive statistics, figures, and tables were used to present the data. Shapiro-Wilk test was done to check the normality of data. To assess model fitness, the Hosmer-Lemeshow test was also used. The data were presented as median (range) for data that were outside the normal distribution. For analysis of categorical data, we used the Chi-square test. Bivariable and multivariable logistic regression were used to know the association of each factor with the outcome variables. From the bivariable logistic regression analysis, factors with *p*-values of <0.2 were entered into multivariable regression analysis. In multivariable analysis, a *p-*value of <0.05 was taken as statistically significant with a 95% confidence interval.

## Result

In our study, 443 patients were included with a 95.7% response rate. Among the study participants, 240(56.6%) were male. The median and interquartile ranges of study participants' age and BMI were (43 ± 45) years Vs (22 ± 9.8) kg/m^2^ respectively. The maximum number of study participants 329(77.5%) were between the age category of 18–64 years. Regarding the educational levels of participants, 167 (39.4%) were able to read and write. Others, 86 (20.3%) of patients did not attend formal education. Regarding residency, 244 (57.5%) were living in rural areas (shown in Table 2).

**Table 2:** Socio-demographic characteristics of orthopedics surgical patients in Amhara regional CSHs, in North–West Ethiopia, from January 10/ 2021 to April 15 / 2021.

**Variables**	**Category**	**Frequency**	**Percentage**
			**(%)**
Gender	Male	240	56.6
	Female	184	43.4
Age	18-54	177	41.7
	55-64	152	35.8
	>64	95	22.5
BMI	<18.5	242	57.1
	18.5-24.9	152	35.8
	>24.9	30	7.1
Marital status	Single	90	21.2
	Married	167	39.4
	Divorced and widowed	167	39.4
Residence	Urban	180	42.5
	Rural	244	57.5
Educational status	Did not read and write	86	20.3
	Primary school (1-8)	167	39.4
	Secondary School and above	171	40.3
Monthly income	>1627 ETB	57	13.4
	<1627 ETB	367	86.6
Occupation	Government employer	125	29.5
	Farmer	14	3.3
	self employee	79	18.6
	student	126	29.7
	Unemployed	80	18.9

BMI, Body Mass-Index; ETB, Ethiopian Birr.

### Clinical characteristics of patients

The majority 359 (84.7%) of participants were under the category of ASA I physical status. Hypertension was the commonest diagnosed comorbidity in 32 (7.5%) and asthma in 11 (2.8%) of orthopedics cases. The remaining 5.03% were HIV and diabetic patients. Among the study participants, 270(63.68%) patients were done under emergency surgery. Regarding the sites of surgery, 283(66.7%) procedures were performed in the lower extremity. Of the total study participants, 274 (64.6%) patients were done under regional Anesthesia. Regarding preoperative substance use, 28 (6.6%) of cases had a previous history of Chat chewing, 17 (4%) were having a history of smoking and 11 (2.6%) had a history of alcohol intake (shown in Table 3).

**Table 3:** Perioperative clinical characteristics of orthopedics surgical patients in Amhara regional CSHs, Northwest Ethiopia, from January 10/2021 to April 15/2021.

**Variables**	**Category**	**Frequency**	**Percentage**
			**(%)**
ASA status	I	359	84.7
	II	48	11.32
	III and above	17	4.01
History of Substance use	Yes	56	13.2
	No	368	86.8
History of anxiety	Yes	185	43.6
	No	239	56.4
Presence of pain	Yes	379	89.4
	No	45	10.6
Social support	Strong	140	33.0
	Moderate	126	29.7
	Poor	158	37.3
Site of surgery	Upper extremity	122	28.8
	Lower extremity	283	66.7
	Other sites	19	4.5
Fracture type	Open fracture	95	22.4
	Closed fracture	329	77.6
Type of surgery	Emergency surgery	265	62.5
	Elective surgery	159	37.5
Type of Anesthesia	Regional Anesthesia	274	64.6
	General Anesthesia	150	35.4

ASA, American society of Anesthesiologist.

### The magnitude of postoperative depression and associated factors

**Figure 1:**
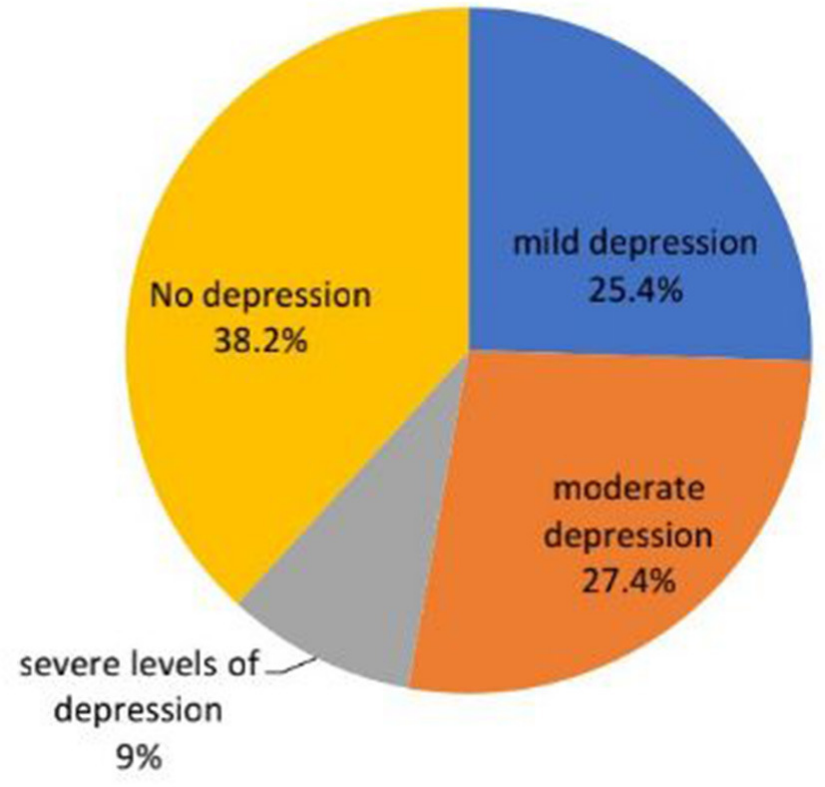
Shows the severity of postoperative depression among adult orthopedics surgical patients in Amhara Regional CSHs, North-West Ethiopia.

Based on our study result, the magnitude of postoperative depression among adult orthopedics surgical patients was 61.8% (95% CI: 56.8–65.7). Among the study participants, 27.4% of patients developed moderate levels of postoperative depression (shown in Figure 1).

Using multivariable logistic regression analysis, factors which had association with postoperative depression were female in gender (AOR: 2.69; 95% CI = 1.46–5.03), Farmer in occupation (AOR: 5.17; 95% CI = 2.22–12.03), having history of previous substance use (AOR: 8.75; 95% CI = 2.86–26.79), history of anxiety (AOR: 3.54; 95% CI = 1.82–6.90), Patients who had moderate to poor social support (AOR: 7.25; 95% CI = 3.89–13.53) and AOR: 2.06; 95% CI = 1.13–3.72), BMI of less than 18.5 kg/m^2^ (AOR: 0.17; 95% CI = 0.08–0.33) and having an open fractures (AOR: 2.42; 95% CI = 1.31–4.44) (shown in Table 4).

**Table 4:** Bivariable and multivariable logistic regression analysis showing factors associated with postoperative depression among orthopedics surgical patients in Amhara regional CSHs.

**Variables**	**Category**	**Post operative depression**		**COR (95% CI)**	**AOR (95% CI)**	***P-*value**
		**Yes**	**No**			
Gender	Male	156(59.5%)	84(51.9%)	1	1	
	Female	106(40.5%)	78(48.1%)	1.37(0.92, 2.03)	2.69(1.46, 5.03)	**0.002**
Occupation	Government employed	90(34.4%)	35(21.6%)	1	1	
	Farmer	11(4.2%)	3(1.8%)	0.54(0.29, 0.98)	5.17(2.22, 12.03)	**<0.001**
	Self employed	46(17.6%)	33(20.4%)	0.47(0.28, 0.79)	1.27(0.53, 3.03)	0.592
	Student	69(26.3%)	57(35.2%)	1.43(0.37, 5.42)	1.43(0.68, 3.06)	0.346
	unemployed	46(17.6%)	34(21.0%)	0.53(0.29, 0.95)	3.66(0.75, 17.92)	0.109
History of substance use	Yes	52(19.8%)	4(2.5%)	9.78(3.46, 27.6)	8.75(2.86, 26.79)	**<0.001**
	No	210(80.2%)	158(97.5%)	1	1	
History of anxiety	Yes	131(50%)	54(33.3%)	2.0(1.33, 3.00)	3.54(1.82, 6.90)	**<0.001**
	No	131(50%)	108(66.7%)	1	1	
Social support	poor	129(49.2%)	29(17.9%)	0.16(0.09, 0.27)	2.06(1.13, 3.72)	**0.018**
	Moderate	75(28.6)	51(31.5%)	0.33(0.19, 0.57)	7.25(3.89, 13.53)	**<0.001**
	Strong	58(22.1%)	82(50.6%)	1	1	
Pain level	Yes	227(86.6%)	152(93.8%)	2.34(1.13, 4.87)	2.14(0.86, 5.27)	0.100
	No	35(13.4%)	10(6.2%)	1	1	
BMI	>24.9	22(8.4%)	8(4.9%)	1	1	
	18.5-24.9	77(29.4%)	75(46.3%)	0.37(0.16, 0.89)	1.48(0.39, 5.69)	0.565
	<18.5 kg/m^2^	163(62.2%)	79(48.8%)	0.75(0.32, 1.76)	0.17(0.08, 0.33)	**<0.001**
Type of fracture	Open fracture	44(16.8%)	51(31.5%)	2.28(1.43, 3.62)	2.42(1.31, 4.44)	**0.005**
	Closed fracture	218(83.2%)	111(68.5%)	1	1	
History of comorbidity	Yes	50(19.1%)	15(9.3%)	2.31(1.25, 4.27)	1.98(0.89, 4.38)	0.091
	No	212(80.9%)	147(90.7%)	1	1	
Type of surgery	Emergency	155(59.2%)	110(67.9%)	1.46(0.97, 2.20)	1.66(0.97, 2.86)	0.064
	Elective	107(40.8%)	52(32.2%)	1	1	

AOR, Adjusted Odd Ratio; BMI, Body Mass Index; COR, Crude Odd Ratio.

## Discussion

Our study aimed to assess the magnitude of postoperative depression and its associated factors among adult orthopedics surgical patients in Amhara regional CSHs. Based on this study, the magnitude of postoperative depression among adult orthopedics patients was 61.8%. Among the factors; being female gender, a farmer in occupation, history of substance use, history of anxiety, moderate to poor social support, BMI of <18.5 kg/m^2^, and having an open fracture was shown to have an association with postoperative depression.

The magnitude of postoperative depression results in our study was similar to a study done in Harare public hospital, in which depression was 59.7% (29). The magnitude of postoperative depression in our study was higher compared with a study done in UK 50% (13), and Nigeria (44.5%) (30). The possible causes of the high prevalence of depression in our setup might be due to lack of specific depression prevention and management protocol in the orthopedics ward, and limited awareness among professionals compared with the above study areas.

Our study result showed a lower magnitude report on postoperative depression compared with a study done in India, which was 87.6% (31), and Vietnam at 66.89% (32). Based on the depression prevalence report from Vietnam, mild depression occurred in 33.8%, moderate depression in 30.4%, and severe depression in 3.7% of cases. Whereas; in our study results the severity of postoperative depression showed that 25.4% of patients developed mild, 27.4% moderate levels, and 9% in severe levels of depression. The possible reason for the lower magnitude report of postoperative depression in our study areas might be the majority of orthopedic surgical cases were not major procedures and were done under regional anesthesia. As different studies showed major procedures and surgeries done under general anesthesia were more likely to develop postoperative depression. This was due to extra depressant drug administration for maintenance of general anesthesia and the consumption of much surgical time during major surgery, which ends up in prolonged quality of recovery and hospital stay.

Our study result showed that female patients were 2.69 times more likely to develop postoperative depression after orthopedics surgery compared with those male patients. In line with our report, a study done in India showed that the risk of developing postoperative depression among female patients was 2.29 times more compared with male patients (10). The possible justification could be due to hormonal effects, rather than ethnicity, race, culture, and other potentially confounding social and economic determinants.

Similarly; our study result showed that orthopedics surgical patients with Job backgrounds of Farmers were 5.17 times more likely to develop postoperative depression compared with those self-employed and unemployed patients. Consistent with our study a study from Norway showed that farmers had higher levels of depression compared with non-farmer professionals (33). The differences in depression levels between farmers and non-farmers could be explained by subjective economic status exerted a significant effect on the psychological distress of rural employed people, and this effect was stronger for the farmers than for the non-farmers (34, 35).

The results of our study also showed that patients with a preoperative history of substance use were 8.75 times more likely to develop postoperative depression compared with orthopedics surgical patients who weren't taking any substances. The reports of our study are supported by a study done in Harare public hospital, and Egypt (29, 36). The possible explanation could be usually substance has numerous chemicals which act on the central nervous system and produce an excitation effect. However; those patients who discontinue intake during admission will result in depression due to withdrawal effects.

The results of the present study showed patients with a history of anxiety were 3.54 times more likely to develop postoperative depression compared with postoperative orthopedics patients who weren't anxious. The above report is supported by a study done in Egypt and showed a positive association between anxiety with post-operative depression (36). The possible explanation for the association of depression with anxiety is due to the suppression of the immune system in depressive disorders may expose the patients to increased rates of postoperative infections and increased mortality from cancer. Postoperative depression is usually associated with cognitive impairment, which may be exacerbated by different levels of anxiety.

Based on our study, patients who had moderate to poor social support were 7.25 and 2.06 times more likely to develop postoperative depression compared with those patients who had strong social support, respectively. This result is supported by studies done in Gurage Zone hospital, Paul's Hospital, and a multi-national community survey study from five European countries (Norway, Finland, England, Ireland, and Spain) hospitals, which showed a positive association between postoperative depression with moderate to poor social support (25, 27, 37). The possible explanation for this association in patients with a strong social support network allows them to gain self-efficacy and self-esteem easier by tolerating and justifying the generation of negative emotions such as depression. Social support can also provide problem-solving strategies to the individual, alleviate the harmful effects of stress experiences, and reduce the importance of the problem. When an individual is under stress, a lack of strong social support will make him/her underestimate the problem and results in enhancing their perceived complications (38).

Our study report also showed orthopedics surgical patients who were underweight (BMI < 18.5 kg/m^2^) were more likely to develop postoperative depression compared with surgical patients who had a BMI of above 18.4 kg/m^2^). The present result was supported by a cross-sectional study done in Australia that showed that postoperative depression and being underweight were positively correlated with a prevalence rate of 24% (39). In many cases, being underweight in adult and elderly orthopedics patients is often associated with the patient's frailty, which may indicate poor quality of life for the patient (40). Another study also suggested that being underweight in adult and elderly patients could be a predictor of various unhealthy conditions such as worse cognitive status or depression, under-nutrition, as well as functional disability (41).

At the same time, this study also showed that patients who had open fractures increased the risks of developing postoperative depression by 2.42 times compared with those surgical patients who had closed fractures. The present results of our study are supported by a study done in the USA and showed a positive correlation between depression with psychological stress and type of fracture (AOR: 4.58; 95% CI, 1.57–12.35) (42). The possible justifications for the association of depression with open fracture could be due to an increase in severity of injury in visible open fracture which maximized the psychological stress, and significant limitation of physical motilities.

## Limitations and strengths of the study

The limitations of this study are, that it was conducted on patients who had a heterogeneous surgical type, it lacks a longer follow-up period, and data were collected on patients which had variable durations of postoperative discharge period. It also lacks sub-group analysis. The strength of this study is that it was conducted in multi-center hospitals with larger sample size. This study is also the first to be conducted in Ethiopia on postoperative depression levels.

## Conclusion

The magnitude of postoperative depression among adult orthopedics patients was found to be high. Due emphasis needs to be given to screening and treatment of depression, especially among patients of the female gender, farmer occupation, moderate to poor social support, history of substance use and anxiety, low BMI, and patients who had an open fracture.

## Data availability statement

The raw data supporting the conclusions of this article will be made available by the authors, without undue reservation.

## Ethics statement

The studies involving human participants were reviewed and approved by Debre Tabor University Ethical Review Committee. The patients/participants provided their written informed consent to participate in this study.

## Author contributions

SS has contributed to the preparation of a proposal, development of a questionnaire, study designing, conceptualization, supervising data collection, data entry, data analysis, data interpretation, and final edition of this manuscript. YF has helped with the supervision of data collection, data analysis, and final output writing and participated in the preparation of this manuscript for submission in this journal. All authors contributed to the article and approved the submitted version.

## Conflict of interest

The authors declare that the research was conducted in the absence of any commercial or financial relationships that could be construed as a potential conflict of interest. The Reviewer DT declared a shared affiliation with the Authors SS and YF at the time of the review.

## Publisher's note

All claims expressed in this article are solely those of the authors and do not necessarily represent those of their affiliated organizations, or those of the publisher, the editors and the reviewers. Any product that may be evaluated in this article, or claim that may be made by its manufacturer, is not guaranteed or endorsed by the publisher.

## Abbreviations

ASA: American Society of Anesthesiologists
BM: Body Mass Index
CSHs: Comprehensive Specialized Hospitals
PHQ: Patient Health Questionnaire.
